# Quantitative relationships in delphinid neocortex

**DOI:** 10.3389/fnana.2014.00132

**Published:** 2014-11-26

**Authors:** Heidi S. Mortensen, Bente Pakkenberg, Maria Dam, Rune Dietz, Christian Sonne, Bjarni Mikkelsen, Nina Eriksen

**Affiliations:** ^1^Research Laboratory for Stereology and Neuroscience, Bispebjerg and Frederiksberg University HospitalsCopenhagen, Denmark; ^2^Research Department, Environment AgencyTorshavn, Faroe Islands; ^3^Department of Bioscience, Institute for Bioscience - Arctic Research Centre, Roskilde, University of AarhusRoskilde, Denmark; ^4^Museum of Natural HistoryTorshavn, Faroe Islands

**Keywords:** neocortical cell number, glia/neuron ratio, neocortical cell density, animal cognition, stereology

## Abstract

Possessing large brains and complex behavioral patterns, cetaceans are believed to be highly intelligent. Their brains, which are the largest in the Animal Kingdom and have enormous gyrification compared with terrestrial mammals, have long been of scientific interest. Few studies, however, report total number of brain cells in cetaceans, and even fewer have used unbiased counting methods. In this study, using stereological methods, we estimated the total number of cells in the neocortex of the long-finned pilot whale (*Globicephala melas*) brain. For the first time, we show that a species of dolphin has more neocortical neurons than any mammal studied to date including humans. These cell numbers are compared across various mammals with different brain sizes, and the function of possessing many neurons is discussed. We found that the long-finned pilot whale neocortex has approximately 37.2 × 10^9^ neurons, which is almost twice as many as humans, and 127 × 10^9^ glial cells. Thus, the absolute number of neurons in the human neocortex is *not* correlated with the superior cognitive abilities of humans (at least compared to cetaceans) as has previously been hypothesized. However, as neuron density in long-finned pilot whales is lower than that in humans, their higher cell number appears to be due to their larger brain. Accordingly, our findings make an important contribution to the ongoing debate over quantitative relationships in the mammalian brain.

## Introduction

Cetaceans are divided into odontocetes (toothed whales) and mysticetes (baleen whales). These two suborders appeared and began to diverge in the early Oligocene, about 30 million years ago (Gingerich et al., 1983). During secondary adaptation to water, cetaceans underwent major transformations in body form and physiology, resulting in large, highly encephalised, and extremely gyrified brains compared to those of terrestrial mammals (Oelschläger and Oelschläger, 2002; Marino, 2008). This is especially true for odontocetes such as the sperm whale (*Physeter macrocephalus*) (~10 kg), which has the largest brain in the Animal Kingdom, but it is not the largest animal alive (Marino, 2004). The cetacean brain differs from the terrestrial mammalian brain in many ways. It has a very high level of gyrification, and like the rest of the whale body, it possesses several traits that indicate adaptation to water. The migration of the blowhole from the front to the top of the head facilitates breathing at the water's surface. This migration has also changed the shape of the brain and the cranial nerve distribution (Oelschläger and Oelschläger, 2009). Cetaceans have been observed to be the most gyrencephalic mammals studied to date, also more than predicted based on comparison with other mammals. This could indicate a morphological alteration of the telencephalon associated with the return to the marine environment (Manger et al., 2012).

Cytoarchitectural organization is very complex (Hof et al., 2005), and does not resemble that of terrestrial mammals; layer I is far more cellular, layer II contains atypical neurons, and layer III contains very large pyramidal neurons (Glezer, 2002; Hof et al., 2005). Cortical layer IV is absent or very poorly developed, and so inputs, outputs, and interneuronal connections are very different than those in other mammals (Glezer et al., 1988; Morgane and Glezer, 1990). The cortical arrangement of functional areas has also changed. The frontal region in the cetacean brain is very modest or even absent (Morgane et al., 1980). Compared to primates, it displays its own unique pattern of differentiation, but it is distinctly laminated and comprises several cortical fields as in other lobes (Hof et al., 2005). Electrophysiological mapping studies have placed both the auditory and visual cortices in the parietal regions of dolphins and porpoises (Supin et al., 1978), whereas they are located in the temporal (auditory) and occipital (visual) regions in terrestrial mammals. The auditory region is located on the suprasylvian gyrus, which adjoins the visual areas in the lateral gyrus and dorsal parietal regions. There is no intervening cortex between the auditory areas and the visual areas or between the visual-auditory areas and the sensorimotor areas (Glezer et al., 1988). The auditory systems are smaller in mysticetes than in odontocetes, but the mysticete visual system contains more axons (Oelschläger and Oelschläger, 2002). The olfactory system is greatly reduced, and in fact is almost absent. This is more obvious in odontocetes than mysticetes (Oelschläger and Oelschläger, 2002).

Odontocetes also show specialized hemispheric independency, such as independent eye movements and closure in beluga whales (*Delhinapterus leucas*) and unihemispheric sleep in Amazon river dolphins (*Inia geoffrensis*) (Mukhametov, 1987), beluga whales (Lyamin et al., 2002), and bottlenose dolphins (*Tursiops truncatus*) (Mukhametov et al., 1988; Ridgway et al., 2006).

The Encephalization Quotient (EQ) is a measure of observed brain size relative to expected brain size (Jerison, 1985). Odontocetes brains are highly encephalized and significantly larger than expected for body size. The EQ of living odontocetes are generally on par with non-human primates. But some odontocetes species belonging to the Delphinidae family contains several species with exceptional high EQs above 4.0, a level of encephalization second only to humans (EQ ~7) (Marino, 2004). This had led to speculations that the large brains of cetaceans could be related to cognitive demands associated with echolocation (Jerison, 1985; Ridgway and Au, 1999) or a response to social forces, because cetaceans display complex social patterns (Payne and Mcvay, 1971; Rendell and Whitehead, 2001) and behaviors such as self-recognition (Delfour and Marten, 2001; Reiss and Marino, 2001), cooperation, and tool use (Krutzen et al., 2005). These capabilities, however, are also possessed by other animals including great apes (Roth and Dicke, 2005), elephants (Roth and Dicke, 2005), and even some birds (Emery, 2006). Yet, other studies show the opposite; that the large cetacean brain is merely an efficient thermogenetic organ that effectively counteracts heat loss to the water (Manger, 2006), and that cetacean intelligence is qualitatively not different to other vertebrates (Manger, 2013; Patzke et al., 2013). Thus, it has recently been argued that mammalian brains should not be scaled equally, as brains of the same size do not always contain similar cell numbers (Herculano-Houzel, 2011b). Rather, the absolute number of neurons, irrespective of brain or body size, may be a better predictor of cognitive abilities (Herculano-Houzel, 2011a).

Previous studies report estimations of the total number of brain cells in both mysticetes [common Minke whale (*Balaenoptera acutorostrata*) (Eriksen and Pakkenberg, 2007)] and odontocetes [harbor porpoise (*Phocoena phocoena*) (Walloe et al., 2010)], but no such estimation has been performed for delphinid species, which are expected to have high numbers of neocortical neurons due to high EQ levels and advanced cognitive abilities. Moreover, neither cetaceans nor any other large-brained species have been found to possess more neocortical neurons than humans. In this study, we studied the brain from the long-finned pilot whale (*Globicephala melas* Traill, 1809), which has an EQ of 2.39 (Marino, 2004). The long-finned pilot whale is a delphinid around 5–6 m long (Desportes et al., 1993) that lives in large matriarchal pods (Ottensmeyer and Whitehead, 2003). It inhabits the deep waters of the North Atlantic ranging from the North Atlantic Ocean from Ungava Bay, Disko in Western Greenland, 68°N in Eastern Greenland, Iceland, the Faroe Islands, and Norway, south to North Carolina, the Azores, Madeira, and Mauritania, including the Western Mediterranean (Rice, 1998). They feed on squid and other prey normally found down to 600 m (Baird et al., 2002), and they do not usually dive as deep as other pelagic species such as beaked whales or sperm whales (Heide-Jørgensen et al., 2002). Echolocation is used for navigation and fouraging, and sounds of the long-finned pilot whale range from to 3–18 KHz (Busnel and Dziedzic, 1966). Audiogram shows best hearing from 11 to 50 KHz (Pacini et al., 2010). Like other delphinids, the long-finned pilot whale seems to see well in both air and water (Herman et al., 1975; Waller, 1992).

Using the optical fractionator, we estimated the total number of neocortical cells in the long-finned pilot whale, and found that long-finned pilot whales have the highest number of neocortical cells of any species studied to date, including humans. These numbers are discussed. We also quantified the auditory and visual cortices (both cell numbers and volume), and compared them to other mammals with different brain and body sizes.

## Materials and methods

**Figure 1:**
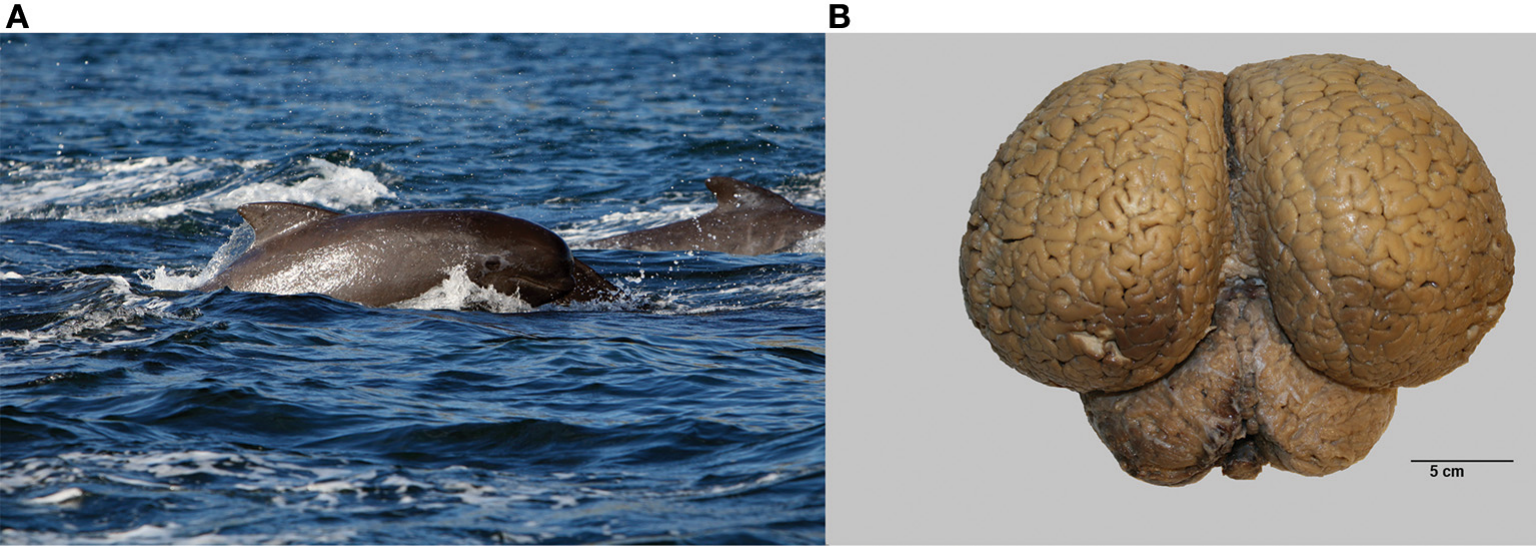
**The long-finned pilot whale (*Globicephala melas*) and its brain**. **(A)** Image of a long-finned pilot whale swimming near the Faroese Islands (copyright Farophoto Inc.). **(B)** Representative image of a long-finned pilot whale brain.

Brains from 10 long-finned pilot whales from the Faroe Islands were used in this study (Figures 1A,B). The brains were collected as part of a local hunt overseen by local authorities and the North Atlantic Marine Mammal Commission (NAMMCO). All animals were healthy and classified as juveniles or adults depending on sexual maturity (Desportes et al., 1993; Martin and Rothery, 1993). Brains were kept in 4.5% formalin for 4 months. A single hemisphere from each animal was chosen at random for analysis. Characteristics of individual animals are shown in Table 1. We were interested in three different brain regions; the entire neocortex, the primary auditory cortex and the primary visual cortex.

**Table 1:** **Characteristics of individual animals used in this study**.

**Individual**	**Hemisphere**	**Sex**	**Length (m)**	**Sexual maturity**	**Total brain weight (g)**
1	Right	M	3.2	Juvenile	3062
2	Left	F	2.7	Juvenile	3142
3	Left	M	3.4	Juvenile	3272
4	Left	M	3.0	Juvenile	3487
5	Left	M	4.6	Juvenile	3705
6	Left	M	4.3	Juvenile	3858
7	Right	F	4.9	Adult	3473
8	Left	F	4.8	Adult	3665
9	Left	M	5.8	Adult	3800
10	Left	M	5.6	Adult	4618

### Cortical mapping

**Figure 2:**
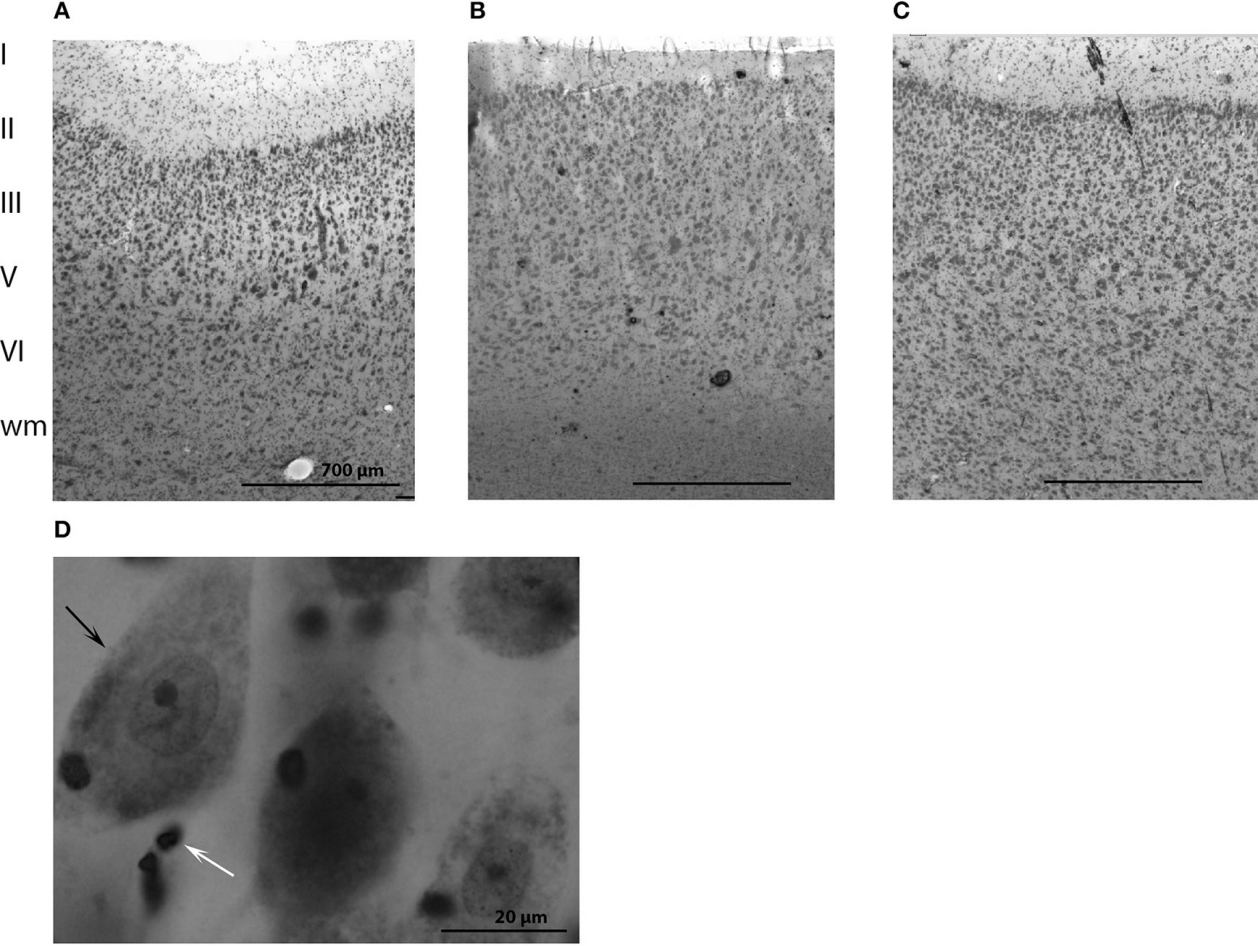
**Anatomy of the long-finned pilot whale brain**. Laminar organization in the three regions of interest (not the especially difference in cell density) in **(A)** the entire neocortex, **(B)** the primary auditory cortex and **(C)** the primary visual cortex. **(D)** Different brain cells. Note the large difference in size of neurons (black arrow) and glial cells (white arrow).

The cytoarchitecture of the primary visual and primary auditory cortices resembled that found by Furutani in the striped dolphin (*Stenella coeruleoalba*), the Risso dolphin (*Grampus griseus*), and the bottlenose dolphin (Furutani, 2008) and harbor porpoise (Walloe et al., 2010) (see, Figures 2A–C for cytoarchitecture in the three brain regions of interest in the long-finned pilot whale brain). Figures 2A–C shows that there is a difference in laminar pattern between the entire neocortex (Figure 2A), the primary auditory (Figure 2B), and visual cortices (Figure 2C). However, it was not possible to identify the subdivisions of neocortex using distinct cytoarchitectural features as described by Hof and Van Der Gucht (2007) in the humpback whale (*Megaptera novaeangliae*) or the presence of well-visible cellular modules as seen in layer II in the occipital lobe of the humpback whale. We therefore, relied on macroscopic identification of primary auditory and visual cortices. Thus, the cortical structure of the long-finned pilot whale was assumed to follow that of other odontocetes (e.g., bottlenose dolphin, harbor porpoise), with primary auditory cortex on the suprasylvian gyrus, between the suprasylvian sulcus and the lateral sulcus, and primary visual cortex on the lateral gyrus between the lateral sulcus and entolateral sulcus in the parietal region (Ladygina et al., 1978; Supin et al., 1978; Morgane and Glezer, 1990). These two cortical areas were marked on the dural surface by different colored tissue dye (CANCER Diagnostics) for later identification.

### Stereological cell counting

**Figure 3:**
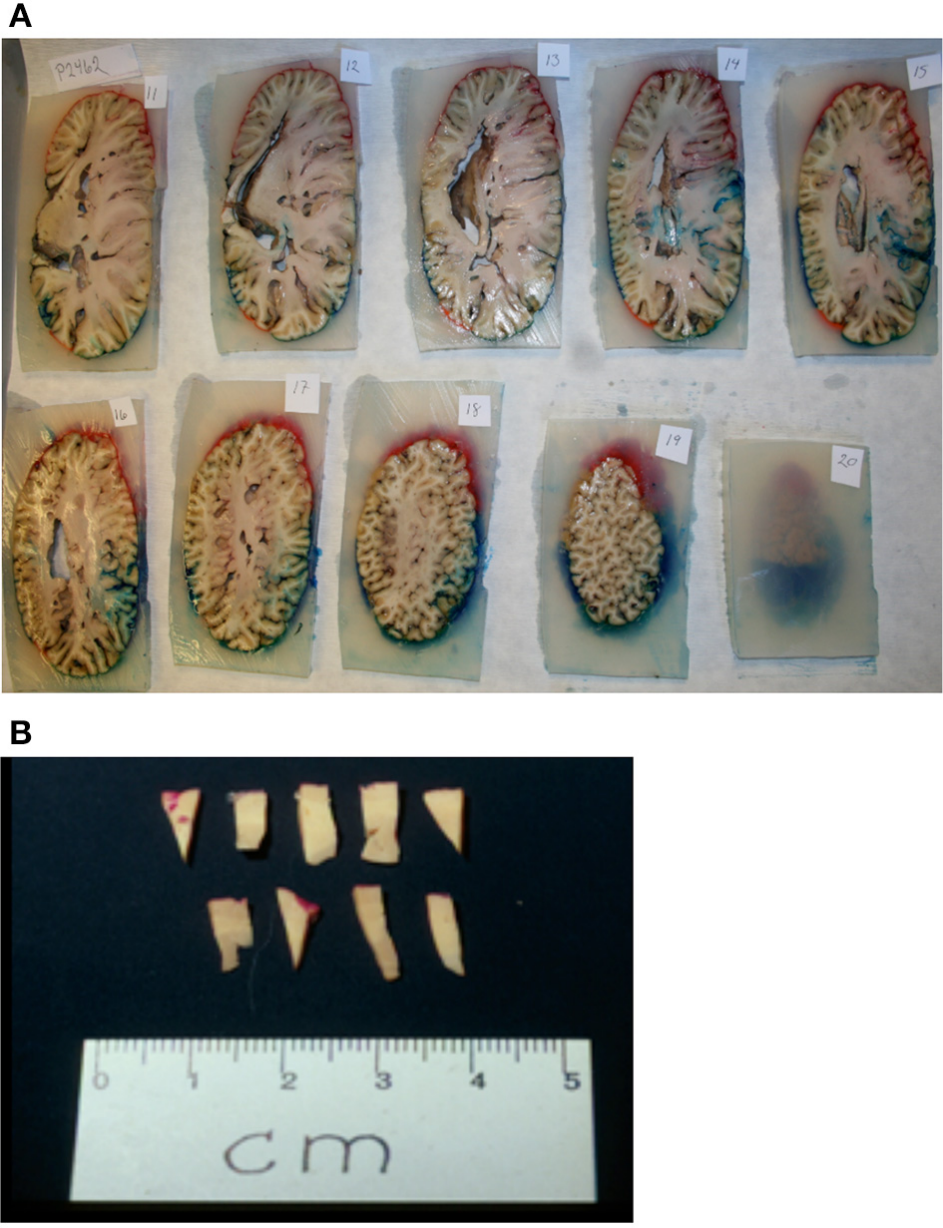
**Sampling procedure**. **(A)** After coloring and embedding in agar, the brain was coronally cut into 1 cm thick consecutive slabs. **(B)** Tissue wedges were sampled from every second slab using SURS. Each wedge was cut into bars, leaving approximately 40 bars. From these 40 bars, 10 bars were subsampled from each region (neocortex, auditory cortex, visual cortex), and embedded in historesin.

Single hemispheres were cut into 1 cm-thick slabs and embedded in agar (Figure 3A). The volume of the three brain regions (*V_ref_*) was calculated using a point-counting grid and the following equation (Gundersen and Jensen, 1987):

$$V_{ref}=t•a\left(p\right)•\sum P$$

where *V_ref_* is the total volume of each brain region, *t* is the thickness of the slab, *a*(*p*) is the predetermined and constant area per point (mm^2^), and ∑*P* is the number of points hitting the region of interest (ROI). A point-counting grid was randomly placed on each slap, and all points hitting the ROI were counted. The average number of slabs was 18.1 (range: 16–20), and the average number of points hitting the entire neocortex was 150 (range: 130–163). The average number of points hitting the auditory cortex was 168 (range: 142–201), and 138 (range: 129–150) for the visual cortex.

A combination of Cavalieri's principle and the optical disector technique (Gundersen and Jensen, 1987; West et al., 1991) was applied to cell counting. The cells counted were neurons and glial cells. From the brain slabs neocortical bars (4 mm) were subsampled from the entire neocortex using systematic uniform random sampling (SURS) (Pakkenberg and Gundersen, 1997), resulting in approximately 4–10 bars per slab (Figure 3B). The subsampled bars were embedded in historesin (KULZER). Shrinkage was less than 5% and therefore, was negligible (Dorph-Petersen et al., 2000). From each bar, a 40 μm-thick section was cut and stained with a modified Giemsa stain. After sampling had been performed on the entire neocortex, the primary auditory and visual cortices were processed in the same manner.

The optical disector, a 3D probe, was placed uniformly randomly on each the sections. Counting frames were superimposed on the magnified image of the tissue, and counting took place inside the thick sections using an unbiased counting frame. Different counting frames were used consecutively for neurons (counting frame area 4500 μm^2^) and glial cells (counting frame area 1200 μm^2^), due to higher numbers of glial cells. All cells were counted in a microscope array with a BX-50 Olympus microscope with a 100× oil immersion objective (2000× final magnification) a motorized x-y stage, an electronic microcator (Heidenhain), and a computer running CAST-GRID software (Visiopharm, Hørsholm, Denmark). Cell density was equal to the number of cells counted in all disectors divided by the volume in which they were counted (Gundersen et al., 1988):

$$N_{v}=\frac{\sum Q}{a\left(frame\right)•h_{dis}•\sum P}$$

where *N_V_* is the cell density, ∑*Q* is the number of cells counted, *a*(*frame*) is the area of the counting frame, *h_dis_* is the disector height. The disector height was 20 μm for both cell types. Total cell number was found by multiplying *V_ref_* by *N_v_*. The mean number of counted cells per specimen was 198 for neurons (range: 145–230) and 238 for glial cells (range: 165–481) for entire neocortex; auditory cortex neurons 176 (range: 141–229), and glial cells 185 (range: 133–234); visual cortex neurons 202 (range: 147–265), and glial cells 204 (range: 133–247).

### Counting criteria

Cells were counted in all layers of cortex from the pial surface to the gray/white transition (Figures 2A–C). Both neurons and glial cells were estimated in the selected brain regions, but we did not distinguish between different types of neurons or glial cells. Neurons were identified as having a clearly defined nucleus with a pale surrounding cytoplasm and a dark, centrally located nucleolus. Glial cells were identified by their smaller size and their lack of cytoplasm. The nucleolus was easily identified in most of the glial cells, and the nucleolus was chosen as the counting item for both neurons and glial cells (Figure 2D). If a nucleolus could not be identified in neurons, the nucleus was used as the counting item. If the cell had more than one nucleolus the most centrally located was used as counting item.

### Statistical analysis

The precision of each estimate was determined by the coefficient of error CE (Gundersen and Jensen, 1987). CE is a function of the noise effect, also known as the point counting variance, and the SURS variance for sums of areas, ∑*a* (Gundersen et al., 1999). The noise effect is the uncertainty that comes from point counting, ∑*P*:

$$Noise=0.0724•\frac{b}{\sqrt{a}}•\sqrt{n•\sum P}$$

where *n* is the number of sections, *b*/√*a* is the average profile shape (found from eyeballing the nomogram from Gundersen and Jensen (1987).

SURS variance for the sum of areas is the uncertainty of sampling between sections, because repeated estimates based on different sections may vary:

$$Var_{SURS\sum a}=\frac{3\left(P_{i}•P_{i}-Noise\right)-4\left(P_{i}•P_{i+1}\right)+\left(P_{i}•P_{i+2}\right)}{240}$$

where *P_i_* is the number of points counted on one section, *P*_*i*+1_ is the number of points counted on the next section. The total sampling variance, *CE*(Σ*P*), is estimated from:

$$CE\left(\sum P\right)=\frac{\sqrt{VAR_{Noise}+VAR_{SURS\sum a}}}{\sum P}$$

Differences between individuals were calculated as the coefficient of variation (CV) using this formula:

$$CV=\frac{SD}{mean}$$

As a rule *CE* is considered optimal, when it is approximately one-half or less of the observed inter-individual variance (*CV_obs_*):

$$\frac{CE^{2}}{CV_{obs}^{2}}\leq 0.5$$

Then the variance is dominated by the biological variance between subjects.

Because data were not normally distributed, Mann–Whitney *U*-tests were used to test for sex differences in cell number, cell density, and neocortical volume. We also wished to investigate the sexual dimorphism within the maturity groups (juvenile or adult), and for this purpose we used a Kruskal–Wallis equality-of-populations rank test. Spearman Rank Sum Correlations were used to assess relationships among body length, neocortical volume, number of cells, and brain weight. Wilcoxon's Sign Rank test was used to test for differences between volumes of auditory and visual cortices. Statistical analysis was performed using Stata 12.1 software (StataCorp LP, USA). Statistical significance was set at *P* < 0.05.

## Results

**Figure 4:**
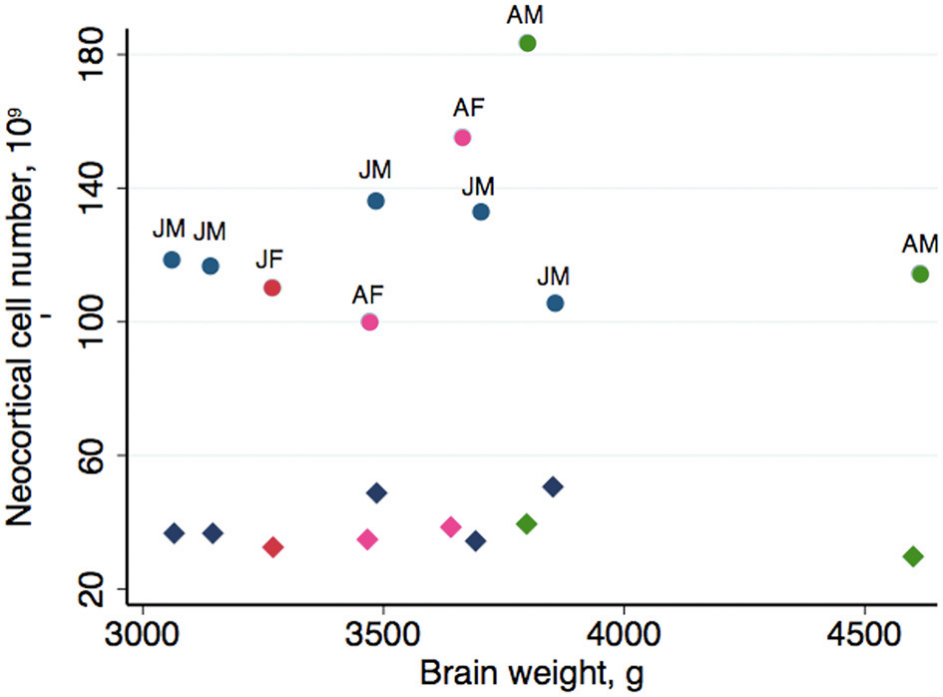
**Neocortical cell number in individual long-finned pilot whales**. Neuron numbers range from 29.1–46.3 × 10^9^, and glial cells range from 99.9–183 × 10^9^. Diamonds indicate neurons, circles indicate glial cells. Abbreviations: *AF*, adult females (pink), *AM*, adult males (green), *JF*, juvenile females (dark red), *JM*, juvenile males (blue).

**Figure 5:**
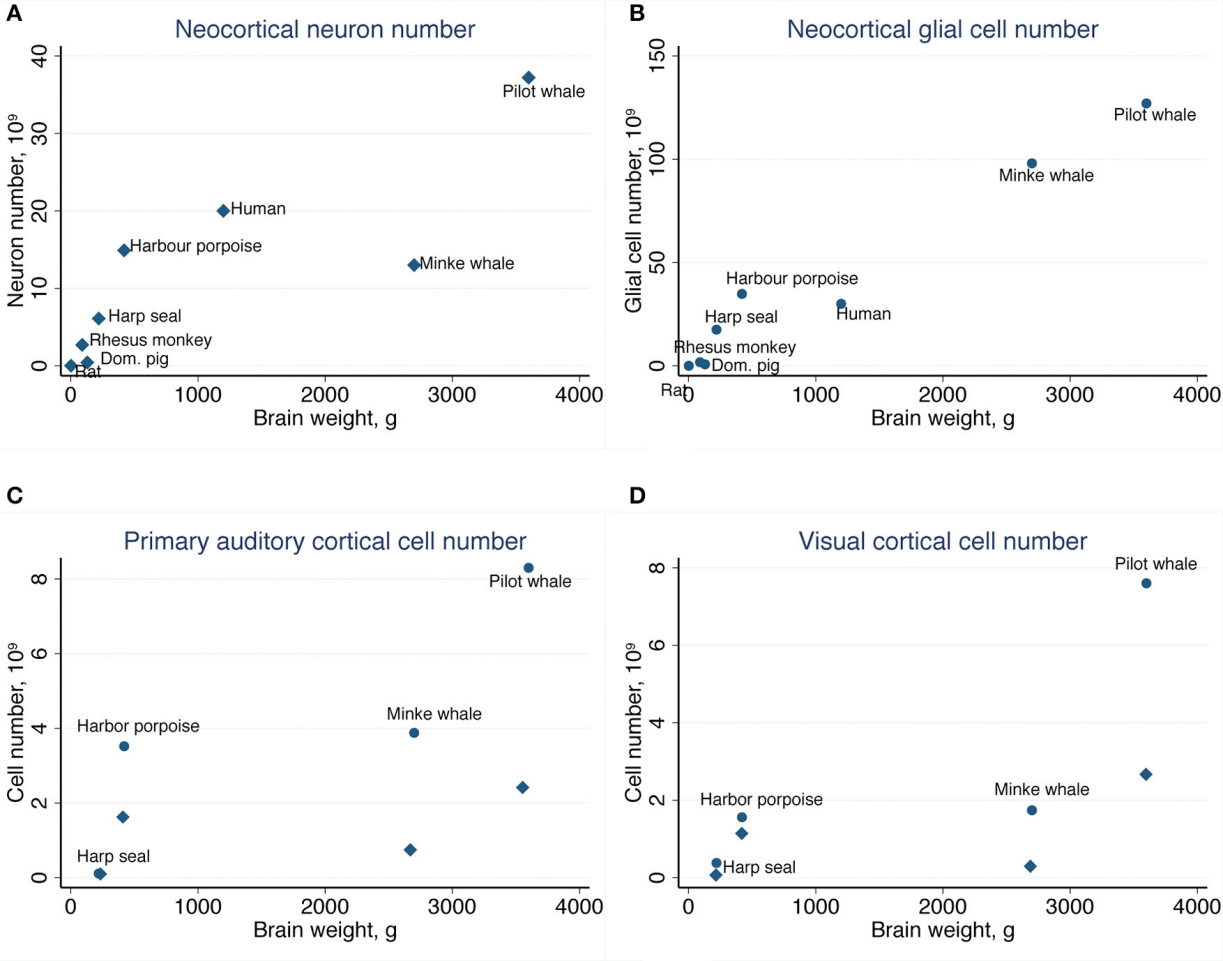
**Comparative analysis of the long-finned pilot whale neocortex**. **(A)** The long-finned pilot whale is estimated to have the highest number of neocortical neurons than any mammal studied to date, with almost twice as many neurons as humans, as well as **(B)** the highest number of neocortical glial cells. Similar findings were observed for the **(C)** primary auditory cortex and **(D)** visual cortex. Diamonds indicate neurons, circles indicate glial cels.

Table 2 summarizes the mean cell numbers found in the long-finned pilot whales, and Figure 4 shows the number of neurons and glial cells in the entire neocortex in each individual animal. We found that, on average, long-finned pilot whales possess 37.2 × 10^9^ neocortical neurons and 127 × 10^9^ neocortical glial cells, with a glial cell to neuron ratio of 3.4/1. Figure 4 shows that the glial cell number was quite variable among the ten individuals (range: 99.9–183 × 10^9^), whereas the neuron number was less variable (range: 29.1–46.3 × 10^9^). It was estimated that long-finned pilot whales have an average of 2.3 × 10^9^ neurons and 8.3 × 10^9^ glial cells in the auditory cortex, and 2.3 × 10^9^ neurons and 7.6 × 10^9^ glial cells in the visual cortex. Next, we compared total neocortical cell numbers across different mammals (Figures 5A,B) and cell numbers in two functional cortices across marine mammals (Figures 5C,D) based on existing stereological literature. We found that the long-finned pilot whale has the highest number of neocortical cells estimated to date. In particular, the long-finned pilot whale has almost twice as many neocortical neurons as humans (Pakkenberg and Gundersen, 1997). Long-finned pilot whales also have the highest number of neocortical cells in both auditory and visual cortices compared with other marine mammals.

**Table 2:** **Estimated cell numbers and density in the long-finned pilot whale brain**.

	**Neocortex**	**CE (CV)**	**Auditory cortex**	**CE (CV)**	**Visual cortex**	**CE (CV)**
**MEAN NEURON NUMBER, 10^9^**						
F	36.9	0.08 (0.04)	2.2	0.08 (0.05)	2.1	0.08 (0.18)
M	37.4	0.08 (0.17)	2.4	0.08 (0.25)	2.4	0.08 (0.17)
All	37.2	0.08 (0.14)	2.3	0.08 (0.20)	2.3	0.08 (0.18)
**MEAN GLIAL CELL NUMBER**						
F	124	0.07 (0.23)	8.6	0.08 (0.18)	7.6	0.07 (0.13)
M	128	0.08 (0.21)	8.2	0.08 (0.29)	7.6	0.08 (0.09)
All	127	0.07 (0.20)	8.3	0.08 (0.24)	7.6	0.08 (0.10)
**MEAN DENSITY OF NEURONS, 10^6^/cm^3^**						
F	31.5		25.8		29.8	
M	30.8		28.4		34.1	
All	30.9		27.4		32.3	
**MEAN DENSITY OF GLIAL CELLS, 10^6^/cm^3^**						
F	104		102		109	
M	105		94.6		106	
All	105		97.3		107	
**MEAN VOLUME, cm^3^**						
F	1178	0.04 (0.09)	84	0.03 (0.07)	70	0.04 (0.05)
M	1222	0.04 (0.14)	85	0.03 (0.16)	71	0.04 (0.05)
All	1209	0.04 (0.12)	85	0.03 (0.13)	71	0.04 (0.05)
**GLIAL CELL/NEURON RATIO**						
All	3.4/1		3.6/1		3.4/1	

Furthermore, we found that long-finned pilot whales have a mean neocortical neuron density of 30.9 × 10^6^/cm^3^ and a mean neocortical glial cell density of 105 × 10^6^/cm^3^. Mean neuron density in the auditory cortex is 27.4 × 10^6^/cm^3^ and mean neuron density in the visual cortex is 32.3 × 10^6^/cm^3^. Mean glial cell density in the auditory cortex is 97.3 × 10^6^/cm^3^ and in the visual cortex is 107 × 10^6^/cm^3^.

There was no sexual dimorphism in cell number, neocortical volume, or neuron density and no significant correlations between brain weight and total cell number or between neocortical volume and total neuron number. Likewise, there was no sexual dimorphism within the two maturity groups. There was no correlation (*r*^2^ = 0.6, *P* = 0.08, Spearman) between neocortical volume and total glial cell number. No significant correlations were found between brain weight and cell density or between total neocortical volume and cell density. The volume of the auditory cortex was significantly greater than that of the visual cortex (*P* = 0.018, Wilcoxon), but there were no significant differences in neuron number or glial cell numbers between the auditory and visual cortices.

**Figure 6:**
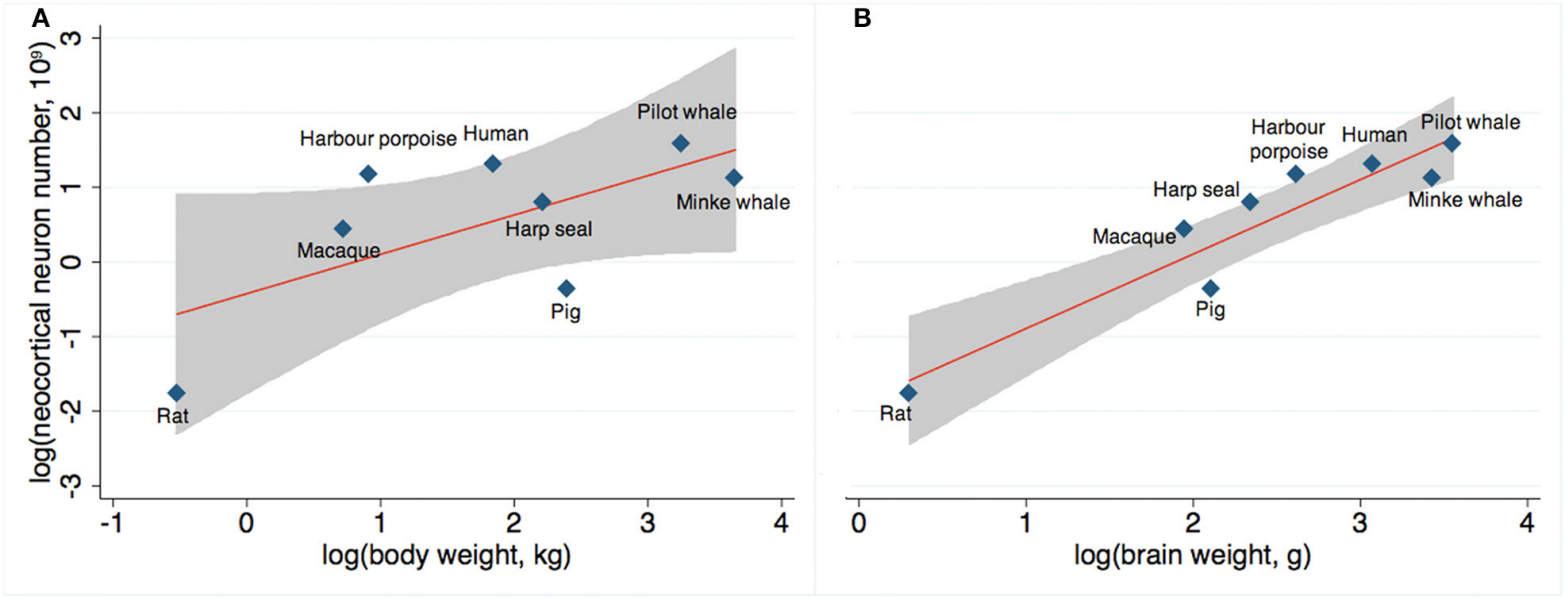
**Expected neuron numbers. (A)** Long-finned pilot whales have a higher than expected number of neocortical neurons relative to body weight, although not as high as that for humans or harbor porpoises, and **(B)** a slightly lower than expected number of neocortical neurons relative to brain weight. Gray shade resembles 95% confidence interval. References: Minke whale (Eriksen and Pakkenberg, 2007), harbor porpoise and harp seal (Walloe et al., 2010), domestic pig (Jelsing et al., 2006), rat (Korbo et al., 1990), Rhesus monkey (macaque) (Christensen et al., 2007), human (Pakkenberg and Gundersen, 1997).

Finally, across mammals, we compared actual and expected neocortical neuron numbers as a function of body weight (Figure 6A) and brain weight (Figure 6B) based on existing stereological literature (Pakkenberg and Gundersen, 1997; Jelsing et al., 2006; Christensen et al., 2007; Eriksen and Pakkenberg, 2007; Walloe et al., 2010). We found that the number of neocortical neurons in long-finned pilot whales exceeds the expected value with respect to body weight, although not to the same extent as harbor porpoises or humans. The number of neocortical neurons in long-finned pilot whales is just below the expected value with respect to brain weight.

## Discussion

The question of dolphin “intelligence” has long been a subject of intense dispute and speculation, ranging from early affirmations (Lilly and Miller, 1961; Lilly, 1965) to recent denial (Manger, 2006, 2013). Across species, neuron number is widely considered to be a major determinant of neural function and, as a consequence, behavior (Williams and Herrup, 1988). Neurons are assumed to be somehow responsible for the evolution of intelligence, as species with larger brains (and thus, more neurons) generally show a greater range and versatility of behavior than species with smaller brains (Jerison, 1985; Marino, 2002).

In this study, we show that the long-finned pilot whale, a species of dolphin, has twice as many neocortical neurons as humans. As neocortical neurons account for 90–95% of all neurons in the cerebrum (Karlsen and Pakkenberg, 2011), this number is approximately equivalent to absolute neuron number. According to the theory that the absolute number of neurons predicts cognitive superiority (Herculano-Houzel, 2011a), the long-finned pilot whale should be cognitively superior to all other species studied, including humans. However, long-finned pilot whales show a higher than expected number of neocortical neurons relative to body weight, but not to the same degree as humans or harbor porpoises. Still, this is an unusually high number of neurons compared to what have been reported recently in other large-brained animals using non-stereological methods (African elephants: 5.6 × 10^9^, gorillas: 9 × 10^9^, orangutans; 8 × 10^9^) (Herculano-Houzel and Kaas, 2011; Herculano-Houzel et al., 2014).

### Cognitive behavior across species

An animal's cognitive capability is widely used as an indicator of mental capacity or intelligence (Roth and Dicke, 2005). Advanced cognitive capabilities are observed in a variety of animals. For example, social transmission of complex songs has been observed in both birds and mysticetes (Payne and Mcvay, 1971; Mundinger, 1980), self-recognition has been observed in several mammals (including three species of dolphins) (Delfour and Marten, 2001; Reiss and Marino, 2001; Roth and Dicke, 2005; Plotnik et al., 2006) as well as in magpies (Prior et al., 2008), and the use of tools has been observed in dolphins (Krutzen et al., 2005), great apes, elephants (Roth and Dicke, 2005), and birds (Roth and Dicke, 2005). The bird brain, however, is structurally very different from the mammalian brain (Reiner et al., 2005), and the number of neurons in birds is much less than that found in mammals (unpublished data). Across animal classes, therefore, the number of neurons is not equal to cognitive capability; rather, these capabilities appear to be cases of convergent evolution (Emery, 2006).

The cognitive capabilities of the long-finned pilot whale (and most other cetaceans for that matter) are unknown, but there have been extensive studies of captive bottlenose dolphins. Most research has revolved around acoustical behavior, with evidence that bottlenose dolphins are capable of understanding and manipulation of symbols, interpretation of images (Herman, 2010), social perception (as described above; e.g., mirror recognition), and numerical cognition (Jaakkola et al., 2005). These skills have been referred to some level of mental capacity or aspect of intelligence (Herman, 2010). However, this has been disputed by Manger, especially since dolphins lack stage 6 (the highest stage) understanding of object performance (Manger, 2013).

The neocortical neuron number of the bottlenose dolphin is unknown, but both long-finned pilot whales and harbor porpoises have an unexpected high number of neocortical neurons, harbor porpoises even to same degree as humans (Figure 6). It is not known whether this high neuron number implies cognitive and/or functional impact, but it is tempting to speculate that these numbers may be related to some advanced cognitive behaviors (here assuming that some parallels can be drawn among delphinid species). However, these behaviors are also seen separately in other mammals with a lower neuron number.

### Glia/neuron ratio

In addition to a high number of neocortical neurons, long-finned pilot whales also have the highest number of neocortical glial cells in any species studied to date. The glia/neuron ratio of 3.4/1 observed here is lower than that observed in Minke whales (7.7/1) (Eriksen and Pakkenberg, 2007), whereas it is higher than that in humans (1.4/1) (Pakkenberg and Gundersen, 1997), and harbor porpoises (2.4/1) (Walloe et al., 2010; Eriksen and Pakkenberg, 2014). Glia/neuron ratios have not been reported for other cetaceans with the exception of bottlenose dolphins (~3/1) and fin whales (*Balaenoptera physalus*) (~5/1) (Oelschläger and Oelschläger, 2002), for which cell counts were not stereologically estimated. Still, these numbers indicate that there is a tendency for increased glia/neuron ratios with greater brain mass. Neurons are increasingly energetically expensive in larger neocortices, and an increased number of glial cells might proliferate to provide metabolic support to neurons. Thus, the absolute number of glial cells in the brain provides an indication of the metabolic demand of neighboring neurons (Sherwood et al., 2006). Minke whales also have very large neuronal perikaryon volume, suggesting that large neurons may require a large number of glial cells (Eriksen and Pakkenberg, 2007, 2014). As glia/neuron ratio may reflect the importance of glia in facilitating neuron growth and hence, neocortical function, it likely plays an important role in cetaceans (Eriksen and Pakkenberg, 2007).

It has been hypothesized recently that an unusually high number of glial cells together with unihemispheric sleep is an effective way to counteract heat loss in cetaceans (Manger, 2006). This would especially apply to deep diving species. As mentioned above all cetaceans studied have shown a very high number of glial cells in their neocortex (Eriksen and Pakkenberg, 2007; Walloe et al., 2010) with the highest glia/neuron ratio is observed in Minke whales (Eriksen and Pakkenberg, 2007). However, Minke whales also have rather large corpus callosi (Ratner et al., 2010), much larger than investigated in odontocete species (Tarpley and Ridgway, 1994; Keogh and Ridgway, 2008), indicating that the specialized hemispheric independency is probably absent in mysticetes (Ratner et al., 2010).

### Functional cortices

Extensive work has been performed on cytoarchitecture in functional corticies (limbic lobe, visual cortex) in a number of odontocetes by Glezer, Morgan and Garey and co-workers (Morgane et al., 1982; Garey and Leuba, 1986; Glezer and Morgane, 1990; Glezer et al., 1993). However, only a couple of studies report actual cell numbers (Poth et al., 2005; Eriksen and Pakkenberg, 2007; Walloe et al., 2010). We are well aware that it is a major limitation that the functional cortices in this study are not delineated using distinct cytoarchitectual indicators. The cell numbers and volume of these cortices must thus be evaluated with caution. However, the outlining on the cortical surface was a copy of the atlas of the bottlenose dolphin (Ladygina et al., 1978; Supin et al., 1978; Morgane and Glezer, 1990), which gives it some validation. Consistent with previous studies, we found that primary auditory cortex volume was larger than visual cortex volume. We also found that long-finned pilot whales have twice as many neurons in the auditory and visual cortices as harbor porpoises (Walloe et al., 2010). Since the density of neurons are around three times lower than in harbor porpoises (neurons: 98.7 × 10^6^/cm^3^, unpublished data), the larger number of neurons in the two cortices in the long-finned pilot whale are most likely due to larger volume. Surprisingly, however, we found no difference in neuron number between the primary auditory and visual cortices in the long-finned pilot whale, even though delphinids rely heavily on audition. Similar results were found by Poth and colleagues, who estimated neuron number per neocortical unit in sensorimotor, auditory, and visual cortices in six species of odontocetes (Poth et al., 2005). Thus, auditory specialization might lie elsewhere in the brain, or odontocetes may rely more heavily on vision than previously assumed (Teilmann et al., 2007; Herman, 2010). However, our finding, though interpreted with caution, might suggest that the high number of neocortical neurons in the long-finned pilot whale is probably not a product of cognitive demands associated with echolocation, as was previously suggested (Jerison, 1985; Ridgway and Au, 1999).

For comparison, using non-stereological methods, it has been estimated that macaques (*Macaca mulatta*) with a brain weight of ~80 g have 5.4 × 10^6^ neurons in the auditory cortex, and baboons (*Papio cynocephalus Anubis*) with a brain weight of ~150 g have 7.1 × 10^6^ neurons in the auditory cortex (Wong et al., 2013).

One other study reports the cell density in the visual cortex of the bottlenose dolphin (Garey and Leuba, 1986), and the neuron density (69.7 × 10^3^/mm^3^) is around twice that of long-finned pilot whale in this study. However, both anterior and posterior end of the lateral gyrus was only reported for a single adult animal, and thus it is hard to compare to our results.

In conclusion, we found that the neocortex of the long-finned pilot whale contains substantially more neurons and glial cells than the neocortex of other large-brained species including humans. As expected, due to their larger brains, odontocetes have substantially more brain cells than mysticetes (Marino, 2002, 2004; Marino et al., 2007). Our results underscore that correlations between cognitive performance and absolute neocortical neuron numbers across animal orders or classes are of limited value, and attempts to quantify the mental capacity of a dolphin for cross-species comparisons are bound to be controversial.

### Conflict of interest statement

The authors declare that the research was conducted in the absence of any commercial or financial relationships that could be construed as a potential conflict of interest.
